# Supplementary figures and images for: Timing of parental depression on risk of child depression and poor educational outcomes: A population based routine data cohort study from Born in Wales, UK
Source: PLoS One. 2021 Nov 17;16(11):e0258966. doi: 10.1371/journal.pone.0258966 (PMC8598047; doi:10.1371/journal.pone.0258966)

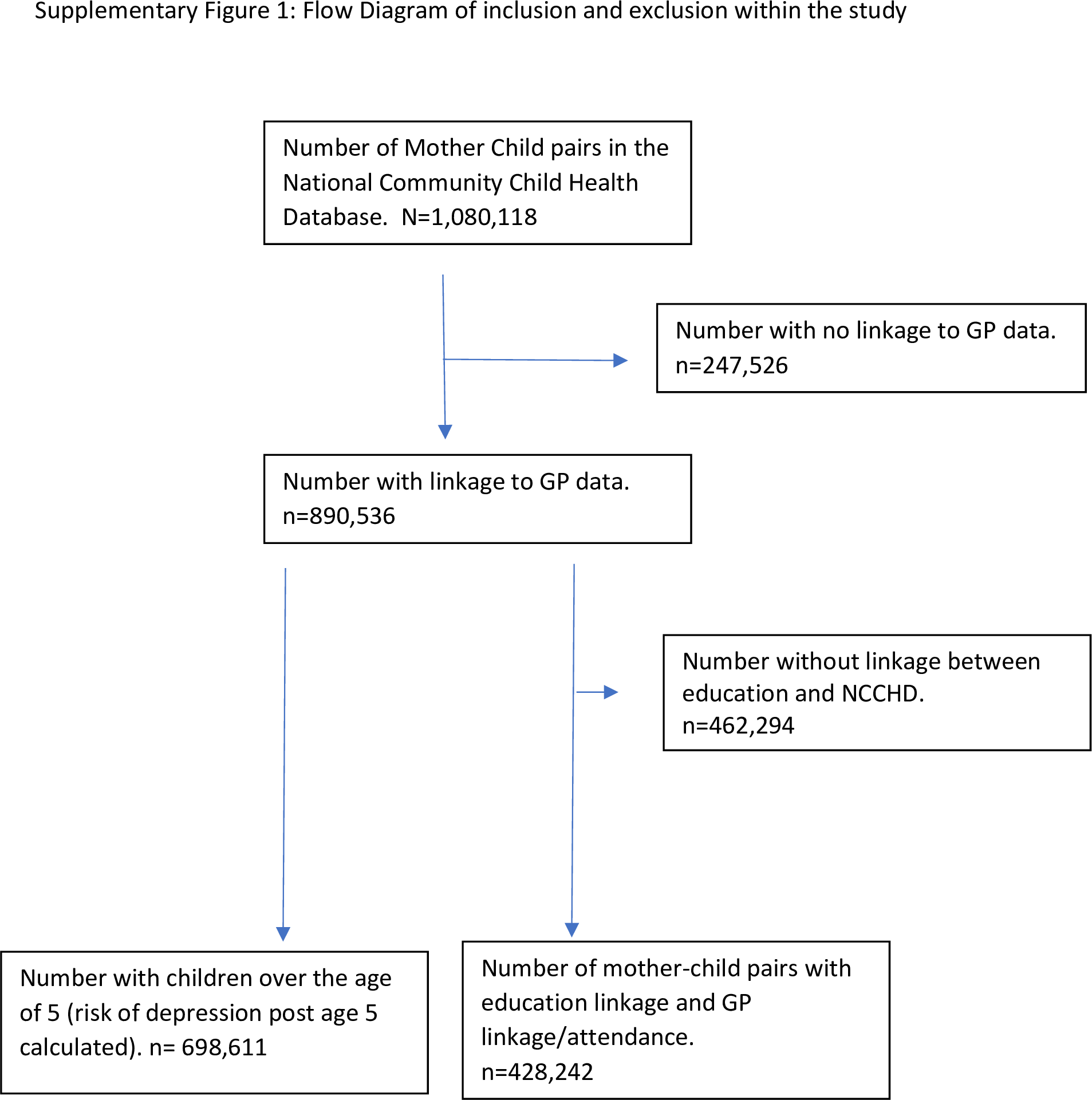

Supplement: S1 Fig — (TIF) [file pone.0258966.s003.tif]
