# Supplementary material for: Timing of parental depression on risk of child depression and poor educational outcomes: A population based routine data cohort study from Born in Wales, UK
Source: PLoS One. 2021 Nov 17;16(11):e0258966. doi: 10.1371/journal.pone.0258966 (PMC8598047; doi:10.1371/journal.pone.0258966)
Supplement: Attachment — Submitted filename: Response to reviewers.docx [file pone.0258966.s004.docx]

Dear Editor,

Thank you very much for your letter outlining points raised during the review process. We have made the following changes:

1. *Please ensure that your manuscript meets PLOS ONE’s style requirements, including those for file naming.*

Done.

2. *a) If there are ethical or legal restrictions on sharing a de-identified data set, please explain them in detail (e.g. data contain potentially identifying or sensitive information) and who has imposed them (e.g. an ethics committee). Please also provide contact information for a data access committee, ethics committee, or other institutional body to which data requests may be sent.*

The data used in this work are provided by the SAIL (Secure Anonymised Information Linkage) Database which is a trusted third party/trusted research environment (TRE) that links identifiable data, is ISO 27001 accredited and provides data following institutional governance approval. To access SAIL data a researcher needs to apply to <https://saildatabank.com/>. The data used in this work cannot be removed from the SAIL gateway as it is potentially identifiable data, but access to the data can be gained by putting in an application to SAIL (<https://saildatabank.com/>)

3. *Please include captions for your supporting Information Files at the end of your manuscript, and update any in-text citations to match accordingly.*

Done

4. *Please review your reference list to ensure that is complete and correct.*

Done

*Reviewers comments:*

Thank you very much for the very positive review of the paper. We have focused on the points that require an amendment or change and detailed the changes made:

*Do the data and analysis full support the claims? If not, what other evidence is required?*

*There are several other confounders that might take part in the educational outcome of children such as any history of learning or intellectual disability or ADHD. The authors might not have this information as part of their dataset, but I think actual HRs might be lower than reported and perhaps even some might be insignificant if other possible confounders are taken into consideration during analysis.*

We have added the following to the discussion “There may also be unmeasured confounders outside of our data remit which have not been included for example access to childcare, parenting style, learning disability and ADHD. The hazards ratios maybe lower than reported if other possible confounders were taken into consideration during the analysis”.

*For the association of leaning outcomes of children and maternal and paternal depression after the birth of the child, looking at the association between the timeline of the depression diagnosis and the children educational achievement afterwards might have been more meaningful.*

We have looked at depression before the child’s birth vs depression during the lifetime of the child. The point raised here focuses on a subgroup of families where we are looking at attainment at age 7, age 11 and age 14 when the mother or the father had depression in the child’s lifetime. We feel calculating a time between depression in the mother and separately for the father/resident male and three repeated measures of for attainment would have been complicated and possibility a separate paper as it was not the main purpose of this research study.

We have added the following to the discussion: Finally, future research would be needed to examine the exact timing of depression for each parent and the relationship with each educational attainment measure for the child.

*On page 8, while reporting the risk effects of paternal depression groups on childhood depression authors state that the ‘confidence intervals overlapped’. Results can still be statistically significant despite overlapping confidence intervals.*

We have added the following to the results: A comparison of those who had depression after (but not before the child’s birth) and those who had depression only before showed a HR 1.16 (95% CI: 0.95 – 1.4), showing no significant difference.

*Reviewer 2:*

1. *Methods: mention the period used to define depression before and after child’s birth. If there were none, then state in the methods sections to give readers some insight.*

The following has been added:

all available GP records were used to identify depression before the birth of the child but all participants need to have a minimum of 2 years of GP records before the birth of the child.

Thank you very much for the advice and help for improving the manuscript.

With very good wishes and thanks

Professor Sinead Brophy on behalf of all the authors.
